# Progression Patterns, Treatment, and Prognosis Beyond Resistance of Responders to Immunotherapy in Advanced Non-Small Cell Lung Cancer

**DOI:** 10.3389/fonc.2021.642883

**Published:** 2021-03-05

**Authors:** Yanjun Xu, Hui Li, Yun Fan

**Affiliations:** Department of Medical Thoracic Oncology, Cancer Hospital of University of Chinese Academy of Sciences, Zhejiang Cancer Hospital, Institute of Cancer Research and Basic Medical Sciences of Chinese Academy of Sciences, Hangzhou, China

**Keywords:** non-small cell lung cancer, immunotherapy, treatment beyond progression, oligoprogression, local radiotherapy

## Abstract

**Introduction:**

Immune checkpoint inhibitors (ICIs) have changed the management of non-small cell lung cancer (NSCLC). However, resistance is inevitable. The disease progression patterns, sequential treatment, and prognosis beyond ICI resistance are not completely understood.

**Methods:**

We retrospectively analyzed stage IV NSCLC patients who underwent ICI treatment at Zhejiang Cancer Hospital between January 2016 and January 2020 and who suffered disease progression after at least stable disease on immunotherapy for more than 3 months (at least two cycles). Oligoprogression and systematic progression were defined as previous reports. The main outcome measures were progression-free survival (PFS), second PFS (PFS2), and overall survival (OS). Survival curves were plotted using the Kaplan-Meier method. The Cox proportional hazards model was used for multivariate analysis.

**Results:**

Totally 1,014 NSCLC patients were administered immunotherapy. Of them, 208 NSCLC patients were included in this retrospective study. The estimated PFS, PFS2 and OS were 6.3 months (95% CI 5.6–7.0 months), 10.7 months (95% CI 10.1–12.7 months), and 21.4 months (95% CI 20.6–26.4 months), respectively. After resistance, 55.3% (N = 115) patients developed oligoprogression, and 44.7% (N = 93) systemic progression. For patients with systemic progression, chemotherapy (N = 35, 37.6%), best supportive care (N = 30, 32.3%), and antiangiogenic therapy alone (N = 11, 11.8%) were the major strategies. A combination of local radiotherapy (N = 38, 33.0%) with continued ICIs was the most common treatment used in oligoprogression group, followed by continued immunotherapy with antiangiogenic therapy (N = 19, 16.5%) and local radiotherapy only (N = 17, 14.9%). For patients with oligoprogression, continued immunotherapy plus local radiotherapy can lead to a significantly longer PFS2 (12.9 *vs.* 10.0 months; *p* = 0.006) and OS (26.3 *vs.* 18.5 months, *p* = 0.001). The PFS2 and OS of patients with oligoprogression were superior to those of patients with systemic progression (PFS2: 13.1 *vs.* 10.0 months, *p* = 0.001; OS: 25.8 *vs*. 19.1 months, *p* = 0.003).

**Conclusions:**

The major progression pattern after acquired resistance from immunotherapy is oligoprogression. Local radiotherapy with continued immunotherapy beyond oligoprogression in responders was feasible and led to prolonged PFS2 and OS in advanced NSCLC patients.

## Introduction

Lung cancer is one of the leading causes of cancer-related mortality worldwide and in China, in which approximately 80% of cases are non-small cell lung cancer (NSCLC). The 5-year overall survival (OS) rate of advanced NSCLC patients is no more than 5%. Recently, the development of immune checkpoint inhibitors (ICIs) targeting cytotoxic T-lymphocyte-associated antigen 4 (CTLA-4), programmed cell death protein-1 (PD-1) or its ligand programmed cell death ligand-1 (PD-L1) has rapidly increased (1). Immune checkpoint blockade has demonstrated impressive effects in advanced NSCLC and prolonged OS (2–4). Thus, ICIs are now widely used in clinical practice and have changed the treatment options and outcomes of advanced NSCLC.

Of note, the tumor response patterns of immunotherapy were found to be different from those of chemotherapy and targeted therapy. Delayed response or stabilization after disease progression (pseudoprogression) has been observed in tumors treated with ICIs, including NSCLC (5). These novel findings have led to the development of immune-based response criteria (6–8), helping in the selection of patients who could benefit from treatment beyond progression (TBP). Many subgroup analyses of clinical trials have been performed to investigate the potential benefit of continuing immunotherapy beyond progression (9–12). In addition, the data of the expanded access program (EAP) and retrospective analyses have also confirmed the benefit of TBP with immunotherapy in NSCLC patients in real-life clinical practice (13, 14). These results indicated that advanced NSCLC patients with pseudoprogression after immunotherapy have a subsequent response and survival benefit from TBP with immunotherapy.

However, acquired resistance is inevitable, and it is uncertain whether patients could also benefit from TBP with immunotherapy plus chemotherapy or other treatment strategies after confirmed disease progression. No prospective studies have focused on the treatment and prognosis after acquired resistance to immunotherapy. Moreover, the disease progression patterns beyond ICIs resistance are not completely understood. For patients who were previously treated with immunotherapy and later showed tumor progression, currently, many patients have fewer treatment options. In clinical practice, at the time of confirmed disease progression, some patients discontinue immunotherapy and initiate a new strategy, such as chemotherapy, antiangiogenesis treatment, local radiotherapy, or best supportive care, while other patients insist on continuing immunotherapy and plus a new strategy.

Although immunotherapy can bring a significant long-term survival benefit in the management of NSCLC, tumors often relapse, known as acquired resistance. The common relapse patterns are unclear. The aim of this retrospective study was to provide detailed information on the effectiveness of ICIs treatment as well as progression patterns, sequential therapy, second progression-free survival (PFS2) and OS after ICIs acquired resistance in patients with advanced NSCLC in real-world routine Chinese clinical practice.

## Materials and Methods

### Patient Eligibility

We reviewed the medical records of NSCLC patients from January 2016 to January 2020 who were administered ICI treatment at Zhejiang Cancer Hospital (N = 1014). A total of 208 stage IV NSCLC patients were identified from a screened population of 1041 patients and enrolled in this study. The inclusion criterias were as follows: 1) patients had pathologically or cytologically proven primary stage IV NSCLC; 2) all the patients benefited from prior immunotherapy with a progression-free survival (PFS) of more than 3 months; 3) patients completed tumor response evaluation for ICI at least once; progressive disease (PD) was confirmed using chest computed tomography (CT), brain magnetic resonance imaging (MRI), and bone scan as well as ultrasound examination and/or CT of the abdomen; 4) patients had at least one measurable lesion and an Eastern Cooperative Oncology Group performance status (PS) score of 0 to 2; 5) patients had epidermal growth factor receptor (EGFR) mutation negative and anaplastic lymphoma kinase (ALK) negative disease; and 6) patients had complete medical records.

### Diagnosis of Oligoprogressive Disease

Oligoprogressive disease is a concept about only a few sites of patients progressed. However, in clinical practice, how to identify oligoprogressive disease remains challenged. Oligoprogressive disease was considered to satisfy the following conditions: 1) one to several distant recurrences (usually one) in one to several organs (usually one); 2) primary site controlled; 3) one to several distant recurrences can be treated with local therapy; 4) no other distant recurrences other than those in 3) (15, 16). In some prospective studies and retrospective reviews, progression patterns were also documented, and oligoprogressive disease was identified as following: 1) progression in the primary site alone, or 2) an asymptomatic solitary site of extra-cranial progression, or 3) three or fewer sites of progression with more than six sites before therapy, or 4) five or fewer sites were progressing (17–21). In our study, oligoprogression was defined as ≤ 2 sites and ≤ 2 lesions of progression and can be treated with local therapy. Systematic progression was defined as ≥ 3 sites and ≥ 3 lesions (usually ≥ 5) of progression.

### Follow-Up

All patients were evaluated for tumor response, PFS, PFS2, and OS. The follow-up rate was 100%. The last follow-up date was July 31, 2020.

### Statistical Analysis

OS was defined as the time from the first cycle of immunotherapy to the date of death or the date of the last follow-up visit for patients who were still alive. PFS was defined as the time from the first cycle of immunotherapy to the first disease progression. PFS2 is defined in the EMA guidance as “time from randomization to objective tumor progression on next-line treatment or death from any cause. In some cases, time on next-line therapy may be used as proxy for PFS” (22). In our study, PFS2 was defined as the time from the first cycle of immunotherapy to the second progression or death. PFS and OS were calculated using the Kaplan-Meier method, and between-treatment differences were assessed by the stratified log-rank test (10% significance level). Hazard ratios (HRs) and 95% confidence intervals (CIs) were estimated based on a stratified Cox model. A p-value of less than 0.05 was regarded as statistically significant. All statistical tests were analyzed using the computer software SPSS version 22.0 (SPSS Inc., Chicago, IL, USA).

## Results

### Patient Characteristics

A total of 1,041 patients were diagnosed with NSCLC and treated with immunotherapy from January 2016 to January 2020 at Zhejiang Cancer Hospital. Patients who received less than two cycles of ICIs, who were lost to follow-up and who did not complete the tumor response assessment were excluded from the study. Patients who had PD as the best response and those who had disease progression at the first assessment of ICI treatment were also excluded from our study. Of the 1,041 patients, 208 (20%) who had a PFS of more than 3 months and later confirmed disease progression were included in the analysis. Among them, 115 (55.3%) patients had oligoprogression, and 93 (44.7%) had systemic progression. The median age of the patients was 61.0 years (range: 32–82 years). The predominant histology of the tumors was squamous cell carcinoma (126/208, 60.6%). A total of 126 patients (126/208, 60.6%) had a smoking history of >= 20 packs of cigarettes/year. Thirty-four (16.3%) patients presented with baseline brain metastasis at the initiation of ICI treatment, and 30 (14.4%) patients had baseline liver metastasis. ICIs were used as first-line treatment in 69 (33.2%) patients, as second-line treatment in 94 (45.2%) patients, and as third-line or later treatment in 45 (21.6%) patients. Sixty-four (30.8%) patients achieved partial response (PR), and 144 (69.2%) had stable disease (SD). A total of 143 (68.8%) patients were treated with ICIs as monotherapy. A greater proportion of patients (68.8%) who achieved PR from immunotherapy developed oligoprogression than systemic progression (31.2%). The patient characteristics are shown in **Table 1**.

**Table 1:** Baseline characteristics of included patients and its correlations with progression model (N=208).

		Total N (%)	Oligo-progression (N=115)		Systemic progression (N=93)		P value
			n	%	n	%	
**Age**	<65	134 (64.4%)	74	55.2	60	44.8	1
	>=65	74 (35.6%)	41	55.4	33	44.5	
**Sex**	Male	171 (82.2%)	100	58.5	71	41.5	0.0672
	Female	37 (17.8%)	15	40.5	22	59.5	
**Smoking**	<20 packs of cigarettes/year	82 (39.4%)	39	47.6	43	52.4	0.0869
	>=20 packs of cigarettes/year	126 (60.6%)	76	60.3	50	39.7	
**ECOG**	0	29 (13.9%)	14	48.3	15	51.7	0.2187
	1	169 (81.3%)	93	55	76	45	
	2	10 (4.8%)	8	80	2	20	
**Pathology**	Squamous cell carcinoma	126 (60.6%)	68	54	58	46	0.6702
	Adenocarcinoma	82 (39.4%)	47	58.3	35	42.7	
**Brain metastases**	Yes	34 (16.3%)	22	64.7	12	35.3	0.2611
	No	174 (83.7%)	93	53.4	81	46.6	
**Liver metastases**	Yes	30 (14.4%)	20	66.7	10	33.3	0.2337
	No	178 (85.6%)	95	53.4	83	46.6	
**Thoracic Radiotherapy**	Yes	70 (33.7%)	41	58.6	29	41.4	0.5559
	No	138 (66.3%)	74	53.6	64	46.4	
**Brain Radiotherapy**	Yes	18 (8.7%)	10	55.6	8	44.4	1
	No	190 (91.3%)	105	55.3	85	44.7	
**Lines of ICI therapy**	1	69 (33.2%)	39	56.5	30	43.5	0.8295
	2	94 (45.2%)	53	56.4	41	43.6	
	3	45 (21.6%)	23	51.1	22	48.9	
**Evaluation of efficacy**	PR	64 (30.8%)	44	68.8	20	31.2	0.0161
	SD	144 (69.2%)	73	50.7	71	49.3	
**Immunotherapy**	Monotherapy	143 (68.8%)	80	55.9	63	44.1	0.8805
	Combination	65 (31.2%)	35	53.8	30	46.2	

ECOG PS, Eastern Cooperative Oncology Group performance score; ICIs, immune checkpoint inhibitors; PR, partial response; SD, stable disease.

### Analysis of the PFS and OS of All the Patients

**Figure 1:**
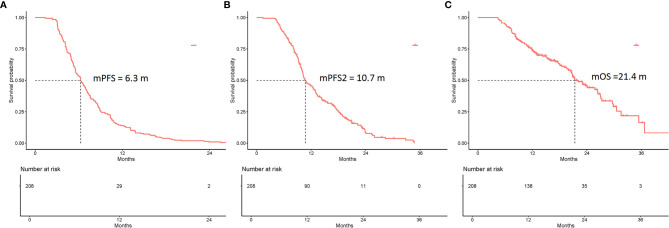
Kaplan-Meier curves of the PFS, PFS2, and overall survival (OS) of all eligible patients (N = 208). **(A)** The mPFS was 6.3 months (95% CI 5.6–7.0 months). **(B)** The mPFS2 was 10.7 months (95% CI 10.1–12.7 months). **(C)** The mOS was 21.4 months (95% CI 20.6–26.4 months).

In total, 1,041 NSCLC patients were administered immunotherapy. Of these, 208 NSCLC patients were included in this retrospective study. The estimated median PFS (mPFS), PFS2, and OS were 6.3 months (95% CI 5.6–7.0 months), 10.7 months (95% CI 10.1–12.7 months), and 21.4 months (95% CI 20.6–26.4 months), respectively (**Figure 1**). Several factors were analyzed to predict PFS with ICIs. In multivariable analysis, pathology [squamous cell carcinoma/adenocarcinoma, HR = 0.68, 95% CI (0.48–0.96); *p* = 0.026], response to ICIs [PR/SD, HR = 1.82, 95% CI (1.28–2.59); *p* = 0.001] and monotherapy or combination therapy [HR = 0.67, 95% CI (0.48–0.96); *p* = 0.027] were independent risk factors for PFS (**Table S1**).

Among all the patients experiencing first PD, the estimated median PFS2 was 10.7 months (95% CI 10.1–12.7 months) (**Figure 1**). The univariate analysis showed that no factors were associated with PFS2. In multivariable analysis, response to ICIs (PR/SD; HR = 1.68; 95% CI 1.16–2.43; *p* = 0.006) was the only independent predictive factor for longer PFS2 (**Table S1**).

The estimated median OS (mOS) was 21.4 months (95% CI 20.6–26.4 months) (**Figure 1**). Multivariate Cox analysis revealed that pathology [squamous cell carcinoma/adenocarcinoma, HR = 0.51, 95% CI (0.32–0.82); *p* = 0.005], response to ICIs [PR/SD, HR = 1.9, 95% CI (1.15–3.12); *p* = 0.012] and monotherapy or combination therapy [HR= 0.55, 95% CI (0.34–0.88); *p* = 0.014] were independent risk factors for OS (**Table S1**).

### Progression Patterns and Sites Beyond Immunotherapy Resistance

**Figure 2:**
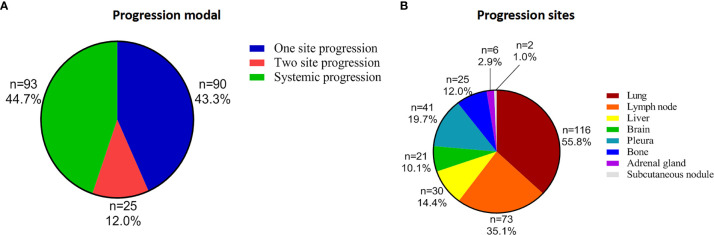
Progression patterns and sites beyond immunotherapy resistance. **(A)** 115 (55.3%) patients developed oligoprogression, and 93 (44.7%) developed systemic progression. Ninety (90/208, 43.3%) patients developed progressive disease at one site. **(B)** The progression sites included the lung (N = 116, 55.8%), lymph node (N = 73, 35.1%), liver (N = 30, 14.4%), brain (N = 21, 10.1%), pleura (N = 41.19.7%), bone (N = 25, 12%), adrenal gland (N = 6, 2.9%), and subcutaneous nodule (N = 2, 1.0%).

The progression patterns and sites of the 208 patients who experienced first disease progression (1st PD) beyond ICIs are shown in **Figure 2** and **Table 2**. Oligoprogression was defined as ≤ 2 sites and ≤ 2 lesions of progression and can be treated with local therapy. Systematic progression was defined as ≥ 3 sites and ≥ 3 lesions (usually ≥ 5) of progression. After resistance to ICIs, 55.3% (N = 115) of patients developed oligoprogression, and 44.7% (N = 93) developed systemic progression (**Figure 2**). Ninety (90/208, 43.3%) patients developed PD at one site. A greater proportion of patients (68.8%) who achieved PR from immunotherapy developed oligoprogression than systemic progression (31.2%) (**Table 1**). The progression sites included the lung (N = 116, 55.8%), lymph node (N = 73, 35.1%), liver (N = 30, 14.4%), brain (N = 21, 10.1%), pleura (N = 41,19.7%), bone (N = 25, 12%), adrenal gland (N = 6, 2.9%), and subcutaneous nodule (N = 2, 1.0%). A total of 85.7% of patients who experienced brain progression exhibited a pattern of oligo-organ progression (**Figure 2**, **Table 2**).

**Table 2:** Progression sites beyond immunotherapy resistance.

Disease site	Total N (%)	Oligo-progression	Systemic progression	P value
**Lung**	116 (55.8%)	50 (43.1%)	66 (56.9%)	0.7249
**Lymph node**	73 (35.1%)	36 (49.3%)	37 (50.7%)	0.4202
**Liver**	30 (14.4%)	15 (50.0%)	15 (50.0%)	0.5662
**Brain**	21 (10.1%)	18 (85.7%)	3 (14.3%)	0.0001
**Pleura**	41 (19.7%)	0 (0.0%)	41 (100.0%)	<0.0001
**Bone**	25 (12%)	16 (64.0%)	9 (36.0%)	0.0577
**Adrenal gland**	6 (2.9%)	3 (50.0%)	3 (50.0%)	1
**Subcutaneous nodule**	2 (1.0%)	2 (100.0%)	0 (0.0%)	0.1980

### Sequential Therapy Beyond Immunotherapy Resistance

The sequential therapies beyond immunotherapy resistance are summarized in **Table 3**. For the patients with systemic progression, chemotherapy (N = 35, 37.6%), best supportive care (N = 30, 32.3%) and antiangiogenic therapy alone (N = 11, 11.8%) were the major treatment strategies. A combination of local radiotherapy (N = 38, 33.0%) on the basis of continued ICI treatment was the most common treatment strategy used in patients with oligoprogression, followed by continued immunotherapy with antiangiogenic therapy (N = 19, 16.5%) and local radiotherapy only (N = 17, 14.9%). Among all patients experiencing 1st PD with oligoprogression, 79 (68.7%) chose to continue immunotherapy beyond progression. In addition, 71 (61.7%) patients with oligoprogression chose local radiotherapy. Only 22 (19.1%) patients with oligoprogression chose systemic chemotherapy.

**Table 3:** Sequential therapy beyond immunotherapy resistance.

Strategy	Total N=208 N (%)	Oligo-progression N=115	Systemic progression N=93	P value
**0**	31 (14.9)	1 (3.2/0.9)	30 (96.8/32.3)	<0.0001
**1**	13 (6.3)	2 (15.4/1.7)	11 (84.6/11.8)	0.0033
**2**	17 (8.2)	17 (100.0/14.9)	0 (0.0)	<0.0001
**3**	40 (19.2)	5 (12.5/4.3)	35 (87.5/37.6)	<0.0001
**1+2**	3 (1.4)	3 (100.0/2.6)	0 (0.0)	0.2548
**1+3**	9 (4.3)	8 (88.9/7.0)	1 (11.1/1.1)	0.0443
**1+4**	28 (13.5)	19 (67.9/16.5)	9 (32.1/9.7)	0.1601
**2+4**	38 (18.3)	38 (100.0/33.0)	0 (0.0/)	<0.0001
**3+4**	16 (7.7)	9 (56.3/7.8)	7 (43.7/7.5)	1
**1+2+4**	13 (6.3)	13 (100.0/11.3)	0 (0.0)	0.0007
**ICI halt**	113 (54.3)	36 (31.9/31.3)	77 (68.1/82.8)	<0.0001
**ICI maintain**	95 (45.7)	79 (83.2/68.7)	16 (16.8/17.2)	<0.0001
**Anti-angiogenic (yes)**	66 (31.7)	45 (68.2/39.1)	21 (31.8/22.6)	0.0114
**Anti-angiogenic (no)**	142 (68.3)	70 (49.3/60.9)	72 (50.7/77.4)	0.0114
**Radiation therapy (yes)**	71 (34.1)	71 (100.0/61.7)	0 (0.0)	<0.0001
**Radiation therapy (no)**	137 (65.9)	44 (32.1/38.3)	93 (67.9/100.0)	<0.0001
**Chemotherapy (yes)**	65 (31.2)	22 (33.8/19.1)	43 (66.2/46.2)	<0.0001
**Chemotherapy (no)**	143 (68.8)	93 (65.0/80.9)	50 (35.0/53.8)	<0.0001

0, best supportive care; 1, anti-angiogenesis; 2, local radiotherapy; 3, chemotherapy; 4, ICI maintain.

### PFS, PFS2, and OS Analyses According to Progression Patterns

**Figure 3:**
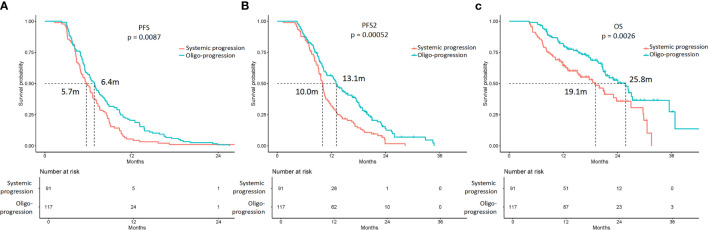
Kaplan-Meier curves of the PFS, PFS2, and overall survival (OS) of patients with oligoprogression and systemic progression. **(A)** The mPFS were 6.4 and 5.7 months in patients with oligoprogression and systemic progression, respectively (*p* = 0.009). **(B)** The mPFS2 were 13.1 and 10.0 months in patients with oligoprogression and systemic progression, respectively (*p* = 0.001). **(C)** The mOS were 25.8 and 19.1 months in patients with oligoprogression and systemic progression, respectively (*p* = 0.003).

The PFS, PFS2, and OS of patients with oligoprogression were superior to those of patients with systemic progression (**Figure 3**, **Table S2**). The estimated mPFS were 6.4 and 5.7 months for patients with oligoprogression and patients with systemic progression, respectively; the difference was statistically significant (*p* = 0.009). The estimated mPFS2 were 13.1 and 10.0 months for patients with oligoprogression and patients with systemic progression, respectively (*p* = 0.001), and the corresponding mOS were 25.8 and 19.1 months (*p* = 0.003).

### PFS2 and OS Analyses According to Sequential Therapy for the Entire Cohort

For the entire cohort, continued ICI treatment beyond 1st PD after ICI treatment can lead to a significantly longer PFS2 (12.9 *vs*. 10.0 months; *p* = 0.006) and OS (26.3 *vs*. 18.5 months; *p* = 0.001) (**Figure S1**). The median duration of ICI treatment was 7.5 months. When separating the patients into two groups according to the ICI treatment length (i.e., ICI >7.5 or ≤ 7.5 months), the mPFS2 and mOS were significantly different. The longer ICI (>7.5 months) treatment group showed superior mPFS2 and mOS compared with the shorter ICI (≤ 7.5 months) treatment group. The estimated mPFS2 values were 16.6 and 8.3 months for the longer and shorter ICI treatment groups, and the mOS were 29.8 and 12.7 months, respectively (*p* < 0.0001) (**Figure S2**).

**Figure 4:**
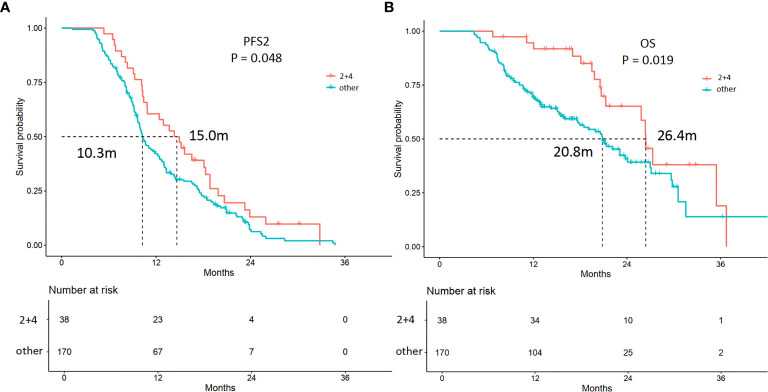
Kaplan-Meier curves of the PFS2 and overall survival (OS) of patients receiving different treatments. **(A)** The mPFS2 were 15.0 and 10.3 months in patients receiving continued ICI plus local therapy (2 + 4) and patients treated with other strategies (*p* = 0.048), respectively. **(B)** The mOS were 26.4 and 20.8 months in patients receiving continued ICI plus local therapy (2 + 4) and patients treated with other strategies (*p* = 0.019), respectively.

Among the 208 patients, 38 (18.3%) patients received continued ICI plus local therapy after resistance. Among these 38 patients, 100% had oligoprogression. In multivariable analysis, continued ICI plus local therapy was a predictive factor for longer PFS2 (*p* = 0.001) and OS (*p* = 0.00) (**Table S3**, **Figure 4**). The estimated mPFS2 values were 15.0 and 10.3 months (*p* = 0.05), and the mOS were 26.4 and 20.8 months (*p* = 0.02) in patients receiving continued ICI plus local therapy (2 + 4) and patients receiving other strategies, respectively (**Figure 4**).

**Figure 5:**
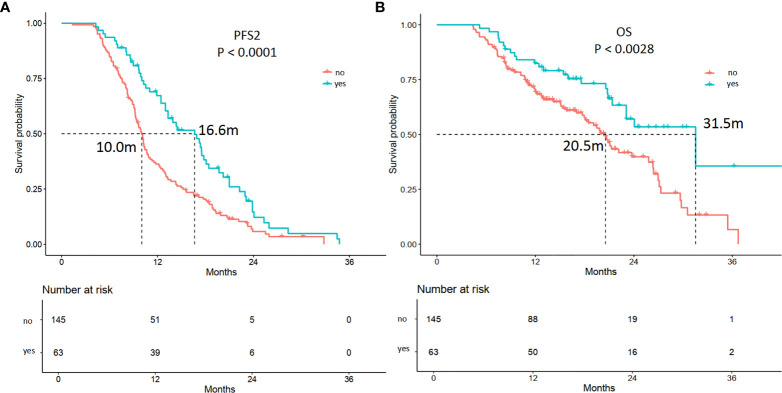
Kaplan-Meier curves of the PFS2 and overall survival (OS) of patients receiving antiangiogenic therapy or not. **(A)** The mPFS2 were 16.6 and 10.0 months in patients receiving antiangiogenic therapy (yes) or not (*p* = 0.00), respectively. **(B)** The mOS were 31.5 and 20.5 months in patients receiving antiangiogenic therapy (yes) or not (*p* = 0.00), respectively.

Among the 208 patients, 66 (31.7%) received antiangiogenic therapy after 1st PD. Forty-five (68.2%) patients had oligoprogression. In multivariable analysis, patients who received antiangiogenic therapy showed longer PFS2 (*p* = 0.00) and OS (*p* = 0.001) (**Table S3**, **Figure 5**). The estimated mPFS2 were 16.6 and 10.0 months (*p* = 0.00), and the mOS were 31.5 and 20.5 months (*p* = 0.00) in patients receiving antiangiogenic therapy and patients who did not receive antiangiogenic therapy, respectively (**Table S3**, **Figure 5**).

### PFS2 and OS Analyses According to Sequential Therapy in Systemic Progression Cohort

In systemic progression cohort (N = 93), 30 (32.3%) patients received best supportive care. Addition of systemic treatment showed a significantly longer PFS [10.4 *vs*. 9.0 m; HR = 0.53, 95% CI (0.34–0.84); p = 0.007] and OS [23.8 *vs*. 10.2 m; HR = 0.3.95% CI (0.17–0.54); p < 0.001] than best supportive care (**Figure S3A**). When further dividing patients into three sub-groups according to different treatment strategies as following: ICI plus anti-angiogenesis or chemotherapy (4 + 1/3), chemotherapy only (3), anti-angiogenesis only (1), the mPFS2 were 10.0, 10.5, and 11.9 months [HR = 1.1, 95% CI (0.58–2.09); *p* = 0.9], and the mOS were 23.1, 23.8, and 12.4 months [HR = 1.36, 95% CI (0.49–3.73); *p* = 0.6], respectively (**Figure S3B**).

### PFS2 and OS Analyses According to Sequential Therapy in Oligoprogression Cohort

In oligoprogression cohort (N = 115), 51 (44.3%) patients treated with continued ICI and local radiotherapy with/without anti-angiogenesis. When compared with patients treated with other strategies, the mPFS2 were 15.6 and 12.2 months [HR = 1.5, 95% CI (0.99–2.27); *p* **=0.053**], and the mOS were 26.4 and 20.8 months [HR = 2.24, 95% CI (1.24–4.05); *p* = 0.006], respectively (**Figure S4A**). When further divided patients into four sub-groups according to different treatment strategies as following: ICI plus local therapy (a1), ICI plus anti-angiogenesis or chemotherapy (a2), local therapy only (a3), and anti-angiogenesis or chemotherapy (a4), the estimated mPFS2 were 15.6, 13.0, 9.2, and 19.2 months [HR = 0.84, 95% CI (0.42–1.7); *p* < 0.001], and the mOS were 26.4, 23.1, 10.8 and NR months [HR = 0.69, 95% CI (0.2–2.35); *p* **<** 0.001], respectively (**Figure S4B**). Thus, subgroup analyses suggested that OS benefit was observed in the continued ICI and local radiotherapy group.

## Discussion

The introduction of ICIs has notably expanded the available therapeutic options for patients with advanced NSCLC. However, there is no standard treatment for these patients after confirmed disease progression or acquired resistance, and their prognosis remains unclear. Our retrospective study provided first-hand data on the disease progression patterns and sites, sequential treatment strategies, and prognosis beyond ICIs acquired resistance in patients with advanced NSCLC in routine Chinese clinical practice at Zhejiang Cancer Hospital.

According to subgroup analyses from prospective trials, continued ICIs beyond disease progression are applicable in approximately 20–50% of patients who experience PD. Continued ICIs lead to subsequent tumor shrinkage or stabilization in 25–80% of PD patients. Moreover, approximately 5–30% of patients may achieve greater and durable survival benefits compared with patients who stop ICIs and change anticancer therapy (9–14, 23–25). In contrast, the innovation of our research is that we excluded patients with pseudoprogression and analyzed acquired drug resistance in NSCLC patients who benefited from immunotherapy for 3 months or more. After resistance from ICIs, 55.3% (N = 115) of patients developed oligoprogression, and 44.7% (N = 93) developed systemic progression. Combination with local radiotherapy (N = 38, 33.0%) on the basis of continued ICIs is the most common treatment used in patients with oligoprogression, followed by continued immunotherapy with antiangiogenic therapy (N = 19, 16.5%). There were 79 (68.7%) patients with oligoprogression who chose to continue ICIs after progression. For patients with oligoprogression beyond 1st PD after ICIs treatment, continued immunotherapy plus local radiotherapy can lead to a significantly longer PFS2 (12.9 *vs*. 10.0 months; *p* = 0.006) and OS (26.3 *vs*. 18.5 months, *p* = 0.001). Currently, established treatment modes after immunotherapy failure are lacking. The continuation of immunotherapy with local radiotherapy beyond progression may be a good choice for patients with oligoprogression as the acquired resistance model. This result must be further validated in population-based clinical research prospectively.

The identification of patients most likely to benefit from continued ICIs beyond progression remains a challenge. Several studies showed that TBP patients had better PSs both at baseline and at progression and had a higher response rate or disease control rate before progression than non-TBP patients (9, 10, 24, 26–28). The present study shows that a greater proportion of patients (68.8%) who achieved PR from immunotherapy before the first progression are more likely to develop oligoprogression. The PFS2 and OS of patients with oligoprogression were superior to those of patients with systemic progression (PFS2: 13.1 *vs*. 10.0 months, *p* = 0.001; OS: 25.8 *vs*. 19.1 months, *p* = 0.003).

Regarding to the frequency of oligoprogressive disease in NSCLC patients under treatment with immunotherapy, Stephan Rheinheimer reported the rate was about 10% to 20% and Antony Mersiades reported the rate was 11% using slightly different criteria. Other studies in melanoma also confirmed the lower rate of oligoprogressive disease after immunotherapy (29, 30). It seems that our conclusion is contrary to their findings. As in our study, totally 1,014 NSCLC patients were administered immunotherapy in our center from January 2016 to January 2020, and screened. Of them, NSCLC patients with imaging evidence of disease progression who benefited from prior immunotherapy with a PFS less than 3 months were excluded from our study. Moreover, most of them were systemic progression patients. In other words, we only included patients with PR and SD (responders) after immunotherapy. This could be the major reason that our conclusion is different to their findings.

Similar evidences were obtained from EGFR mutant NSCLC patients with oligoprogressive disease. A lot of studies suggested that indicating addition of local therapy showed prolonged survival benefit than EGFR-TKI alone in EGFR-mutant NSCLC patients with oligoprogressive disease, including intracranial metastases, primary lesion progression, and liver metastasis (31–34). It is also evident that radiotherapy could kill cancer cells while triggering the release of pro-inflammatory mediators, increasing tumor infiltrating immune cells, and modulating neoantigen expression simultaneously (35). Thus, radiotherapy could enhance immunostimulatory effects and is increasingly viewed as a promising combination strategy with ICIs (36–38).

Nonetheless, there are still no approved criteria for selecting patients who would benefit from continued ICIs treatment beyond disease progression. Patients with better PSs or oligoprogression are more likely to receive ICIs beyond progression. Moreover, the choice of continuing ICIs after resistance is encouraged by the absence of effective treatment strategies. The addition of localized radiotherapy should be considered a useful tool to improve local tumor control, enhancing ICIs efficacy.

The present study possesses intrinsic limitations due to its retrospective design. In addition, the data were collected from a single center, which also influences the clinical applications of our results.

In conclusion, our data suggest that continuing immunotherapy beyond initial progression in addition to local radiotherapy is feasible and effective, especially in oligoprogression patients. Continuing ICIs beyond progression is associated with longer survival in selected patients according to clinical judgment. Future investigations are warranted to identify patients who are most likely to respond after progression according to predictive biomarkers, patient and disease characteristics, and the type of and response to previous treatments both at baseline and at progression. These findings will enhance the personalized approach to clinical decision-making when considering ICIs as a therapeutic choice and continuing immunotherapy beyond progression to maximize its potential benefit.

## Data Availability Statement

The original contributions presented in the study are included in the article/**Supplementary Material**. Further inquiries can be directed to the corresponding author.

## Ethics Statement

The studies involving human participants were reviewed and approved by Ethics Committee of Zhejiang Cancer Hospital. The patients/participants provided their written informed consent to participate in this study.

## Author Contributions

YX and YF contributed to the study conception and design. HL collected the patient samples and interpreted the data. HL performed the statistical analysis. YX was a major contributor in writing the manuscript. All authors contributed to the article and approved the submitted version.

## Funding

This study was supported by the Natural Scientific Foundation of China (no. 81972718), the Natural Scientific Foundation of Zhejiang Province, China (no. LY19H160007), and the Science and Technology Program for Health and Medicine in Zhejiang Province, China (nos. 2021KY541 and 2021KY556).

## Conflict of Interest

The authors declare that the research was conducted in the absence of any commercial or financial relationships that could be construed as a potential conflict of interest.

## Supplementary Material

Supplementary Figure 1 Kaplan-Meier curves of the PFS2 and OS of patients who halted or maintained ICI treatment. **(A)** Continued ICI (ICI maintenance) beyond the first disease progression after ICI treatment can lead to a significantly longer PFS2 than stopping ICI treatment (ICI halt) (12.9 *vs* 10.0 months; *p* = 0.006). **(B)** Continued ICI (ICI maintenance) beyond the first disease progression after ICI treatment can lead to a significantly longer OS than stopping ICI treatment (ICI halt) (26.3 vs. 18.5 months; *p* = 0.001).

Supplementary Figure 2 Kaplan-Meier curves of the PFS2 and OS of patients with longer or shorter ICI treatment. The longer ICI (>7.5 months) treatment group showed superior mPFS2 **(A)** and mOS **(B)** compared with the shorter ICI (≤ 7.5 months) treatment group; the mPFS2 **(A)** were 16.6 and 8.3 months, and the mOS **(B)** were 29.8 and 12.7 months, respectively (*p* < 0.0001).

Supplementary Figure 3 OS between different therapy groups in systemic progression cohort. (A) The mOS were 23.8 and 10.2 months (*p* **<** 0.001) in patients receiving systemic treatment (Yes) or best supportive care (No), respectively. **(B)** Subgroup analysis of OS in patients with different treatment strategies: ICI plus anti-angiogenesis or chemotherapy (4 + 1/3), chemotherapy only (3), anti-angiogenesis only (1), the mOS were 23.1, 23.8 and 12.4 months (*p* **= 0.6**), respectively.

Supplementary Figure 4 OS between different therapy groups in oligoprogressive cohort. **(A)** The mOS were 26.4 and 20.8 months (*p* **=** 0.006) in patients treated with continued ICI and local radiotherapy with/without anti-angiogenesis (a1) and other strategies (others). (B) Subgroup analysis of OS in patients with different treatment strategies: ICI plus local therapy (a1), ICI plus anti-angiogenesis or chemotherapy (a2), local therapy only (a3), and anti-angiogenesis or chemotherapy (a4), the mOS were 26.4, 23.1, 10.8 and NR months (*p* < 0.001), respectively.
