# Supplementary figures and images for: Progression Patterns, Treatment, and Prognosis Beyond Resistance of Responders to Immunotherapy in Advanced Non-Small Cell Lung Cancer
Source: Front Oncol. 2021 Mar 5;11:642883. doi: 10.3389/fonc.2021.642883 (PMC7973268; doi:10.3389/fonc.2021.642883)

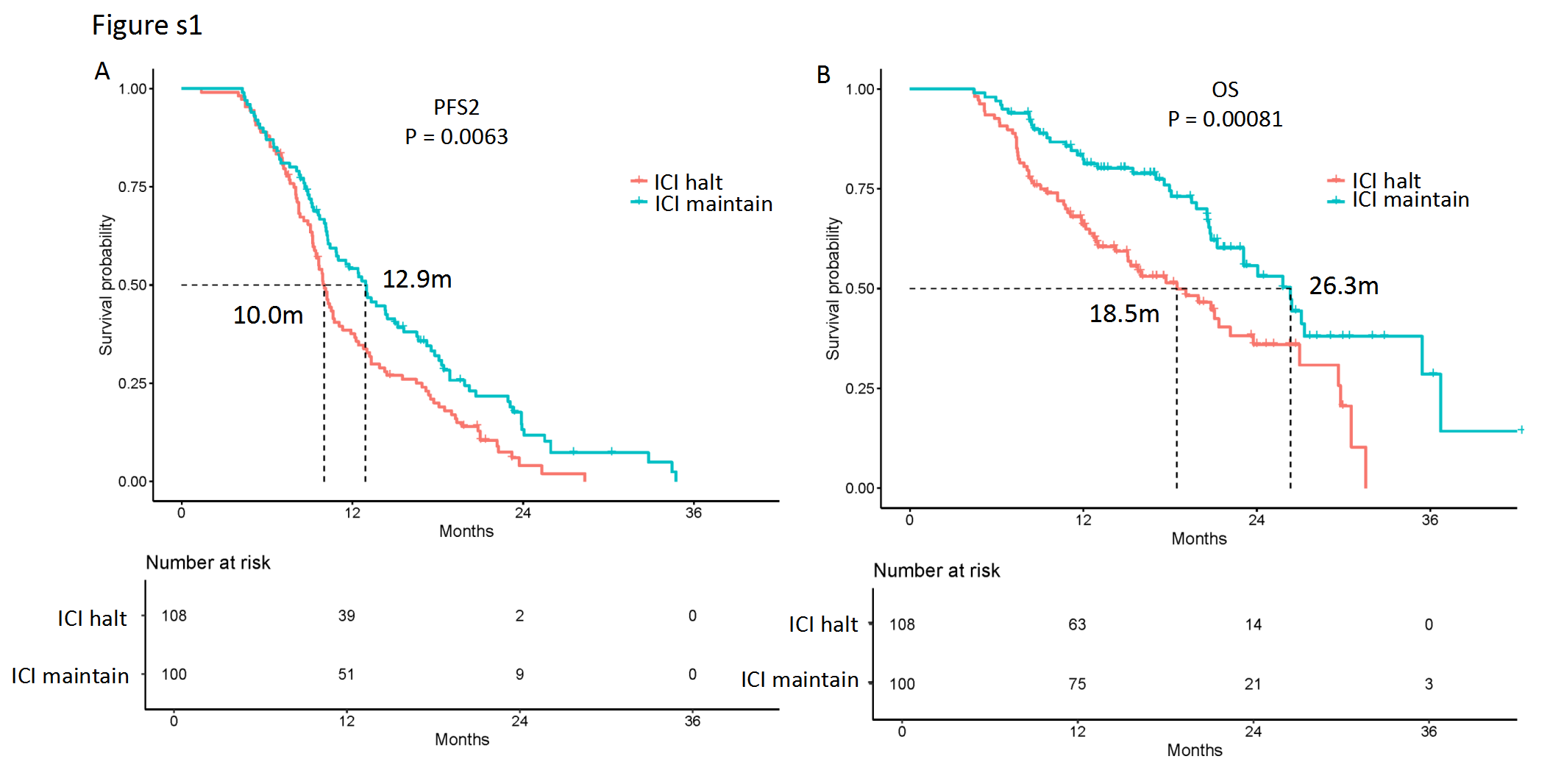

Supplement: Supplementary Figure 1 — Kaplan-Meier curves of the PFS2 and OS of patients who halted or maintained ICI treatment. (A) Continued ICI (ICI maintenance) beyond the first disease progression after ICI treatment can lead to a significantly longer PFS2 than stopping ICI treatment (ICI halt) (12.9 vs 10.0 months; p = 0.006). (B) Continued ICI (ICI maintenance) beyond the first disease progression after ICI treatment can lead to a significantly longer OS than stopping ICI treatment (ICI halt) (26.3 vs. 18.5 months; p = 0.001). [file Image_1.tif]

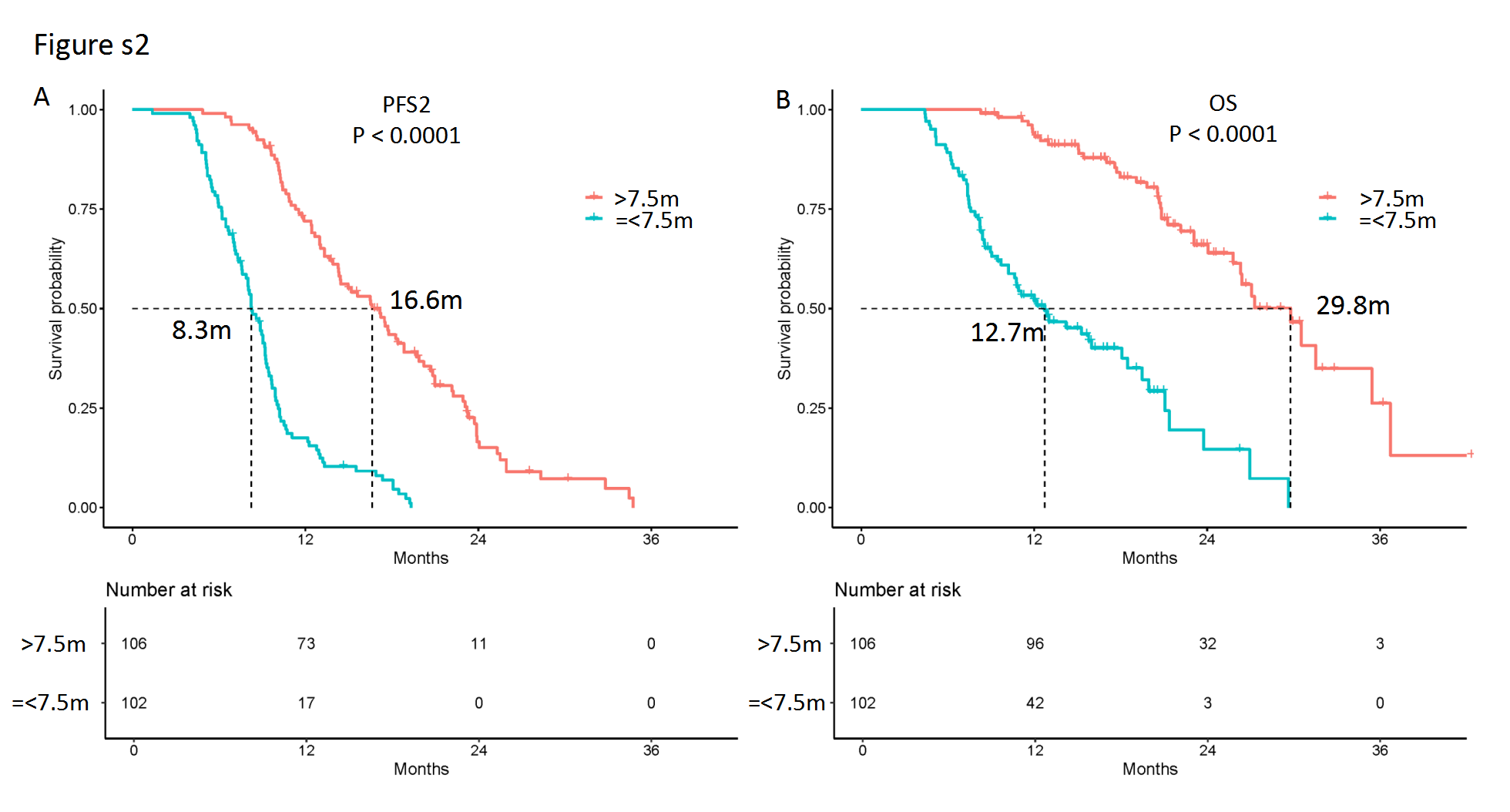

Supplement: Supplementary Figure 2 — Kaplan-Meier curves of the PFS2 and OS of patients with longer or shorter ICI treatment. The longer ICI (>7.5 months) treatment group showed superior mPFS2 (A) and mOS (B) compared with the shorter ICI (≤ 7.5 months) treatment group; the mPFS2 (A) were 16.6 and 8.3 months, and the mOS (B) were 29.8 and 12.7 months, respectively (p < 0.0001). [file Image_2.tif]

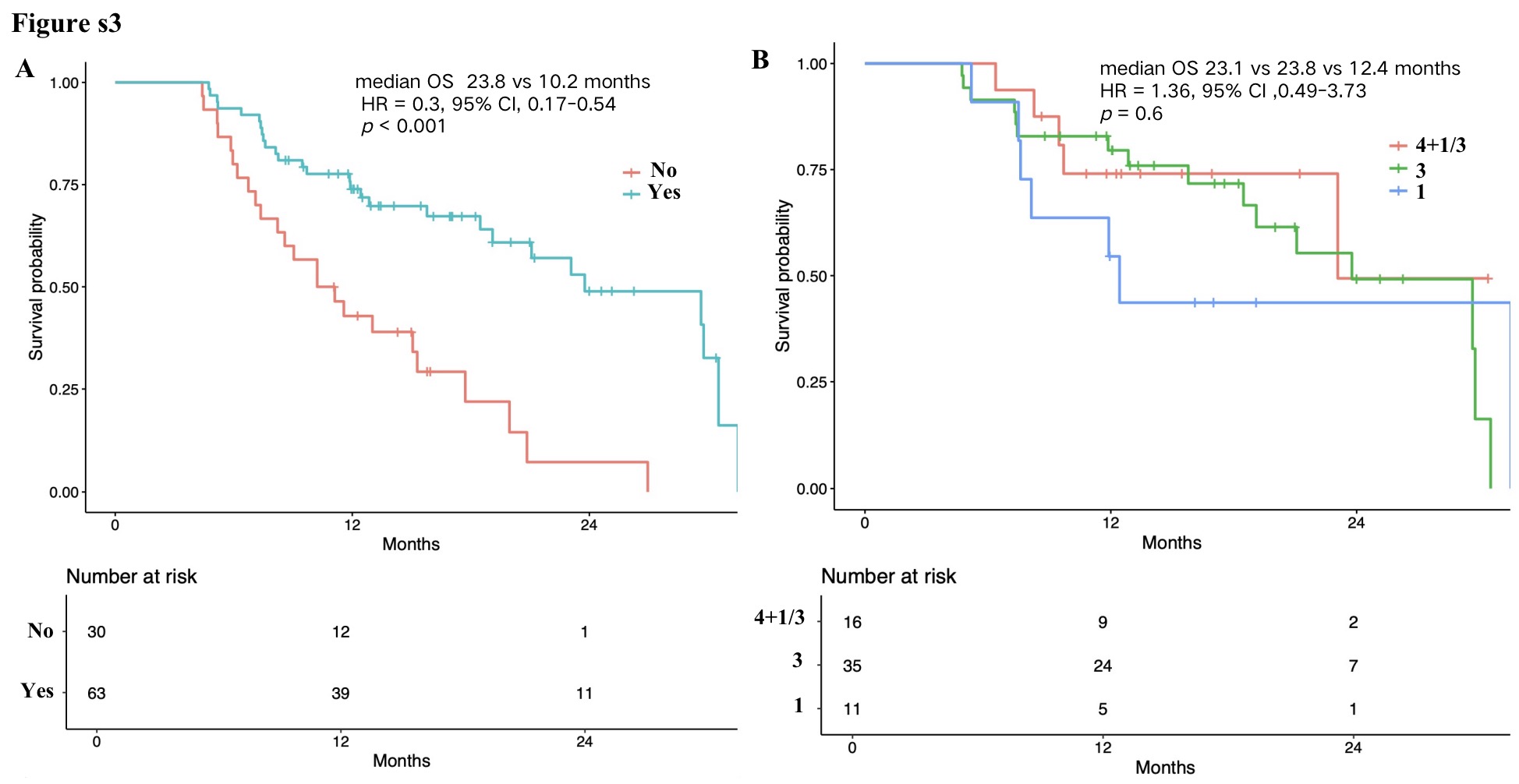

Supplement: Supplementary Figure 3 — OS between different therapy groups in systemic progression cohort. (A) The mOS were 23.8 and 10.2 months (p < 0.001) in patients receiving systemic treatment (Yes) or best supportive care (No), respectively. (B) Subgroup analysis of OS in patients with different treatment strategies: ICI plus anti-angiogenesis or chemotherapy (4 + 1/3), chemotherapy only (3), anti-angiogenesis only (1), the mOS were 23.1, 23.8 and 12.4 months (p = 0.6), respectively. [file Image_3.tif]

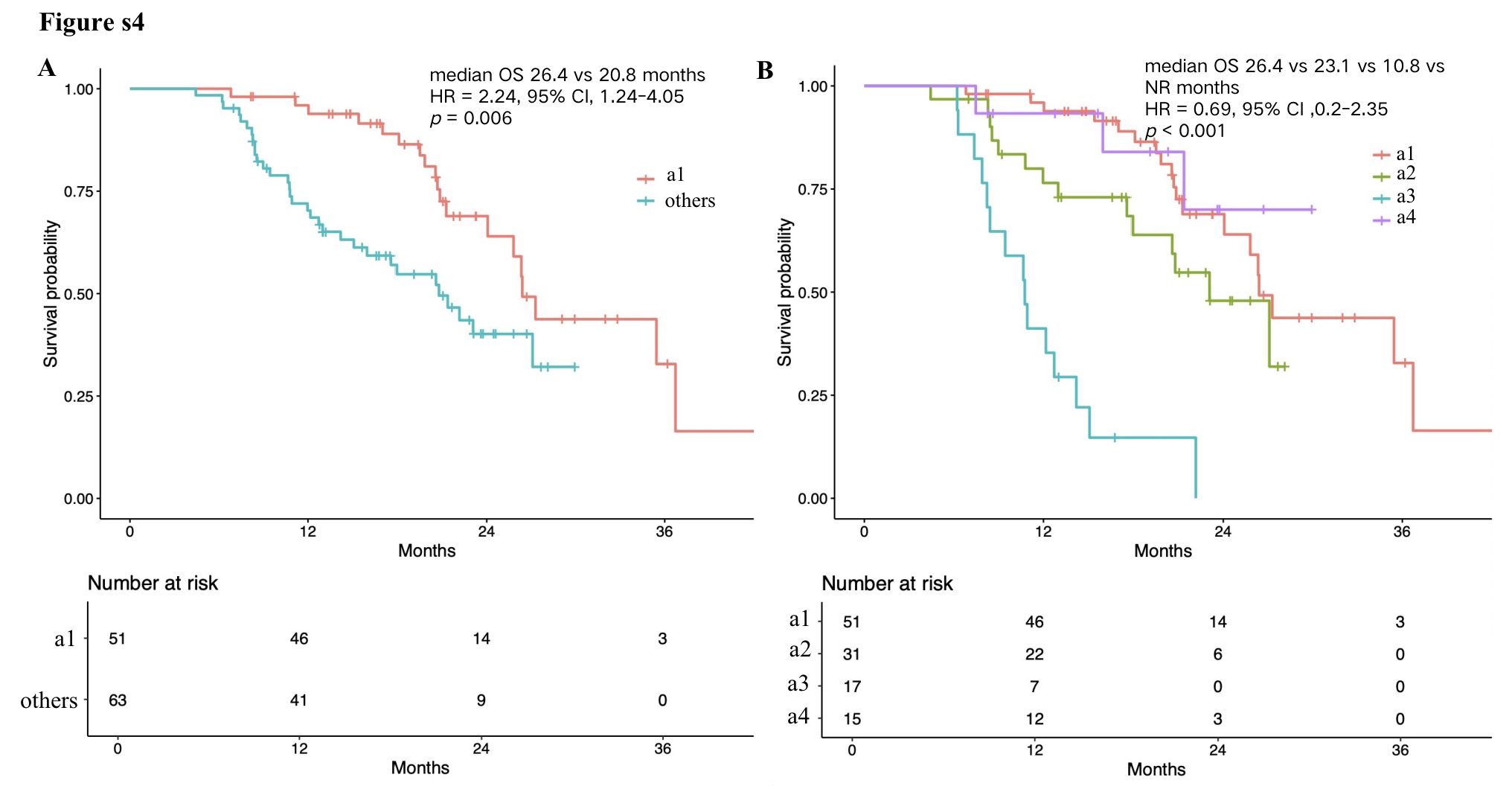

Supplement: Supplementary Figure 4 — OS between different therapy groups in oligoprogressive cohort. (A) The mOS were 26.4 and 20.8 months (p = 0.006) in patients treated with continued ICI and local radiotherapy with/without anti-angiogenesis (a1) and other strategies (others). (B) Subgroup analysis of OS in patients with different treatment strategies: ICI plus local therapy (a1), ICI plus anti-angiogenesis or chemotherapy (a2), local therapy only (a3), and anti-angiogenesis or chemotherapy (a4), the mOS were 26.4, 23.1, 10.8 and NR months (p < 0.001), respectively. [file Image_4.tif]
