# Supplementary material for: Progression Patterns, Treatment, and Prognosis Beyond Resistance of Responders to Immunotherapy in Advanced Non-Small Cell Lung Cancer
Source: Front Oncol. 2021 Mar 5;11:642883. doi: 10.3389/fonc.2021.642883 (PMC7973268; doi:10.3389/fonc.2021.642883)
Supplement: Supplementary file 5 [file DataSheet_1.docx]

**Table s1** Univariate and multivariate analyses of clinical parameters on Progression-Free Survival and Overall Survival (Cox regression) (N=208)

| Factor | Univariate analysis | | | Multivariate analysis | | | |
| --- | --- | --- | --- | --- | --- | --- | --- |
|  | **HR (log rank)** | **95% CI** | **p value** | | **HR (log rank)** | **95% CI** | **p value** |
| Progression-Free Survival |  |  |  | |  |  |  |
| Age (≥65/<65) | 1.06 | 0.8-1.41 | 0.6912 | | 0.98 | 0.72-1.35 | 0.917365 |
| Sex (Male/Female) | 1 | 0.7-1.42 | 0.9837 | | 1.08 | 0.64-1.83 | 0.775726 |
| Smoker (<20 packs of cigarettes/year />=20 packs of cigarettes/year) | 1.08 | 0.8-1.46 | 0.5957 | | 1.06 | 0.67-1.69 | 0.789924 |
| ECOG (0/1/2) | 1.35 | 0.9-2.02 | 0.1467 | | 1.14 | 0.73-1.78 | 0.558103 |
| Pathology (squamous cell carcinoma/adenocarcinoma) | 0.76 | 0.57-1.01 | 0.0584 | | 0.68 | 0.48-0.96 | 0.026386 |
| Brain metastases (Yes/No) | 1.21 | 0.83-1.75 | 0.3201 | | 1.29 | 0.75-2.24 | 0.359525 |
| Liver metastases (Yes/No) | 1.21 | 0.83-1.78 | 0.3223 | | 1.44 | 0.96-2.16 | 0.074964 |
| Thoracic Radiotherapy (Yes/No) | 1.28 | 0.95-1.71 | 0.1012 | | 1.23 | 0.88-1.73 | 0.230675 |
| Brain Radiotherapy (Yes/No) | 1.06 | 0.65-1.73 | 0.8076 | | 0.63 | 0.31-1.27 | 0.19288 |
| Lines of ICI therapy (1/2/3) | 1.13 | 0.83-1.55 | 0.432 | | 0.98 | 0.67-1.45 | 0.93684 |
| Evaluation of efficacy (PR/SD) | 1.64 | 1.21-2.23 | 0.0015 | | 1.82 | 1.28-2.59 | 0.000915 |
| Immunotherapy (monotherapy/combination) | 0.82 | 0.61-1.11 | 0.1943 | | 0.67 | 0.48-0.96 | 0.027089 |
| Progression-Free Survival-2 |  |  |  | |  |  |  |
| Age (≥65/<65) | 1.11 | 0.82-1.51 | 0.4954 | | 1.02 | 0.72-1.45 | 0.89342 |
| Sex (Male/Female) | 1.3 | 0.87-1.93 | 0.2006 | | 1.73 | 0.99-3.02 | 0.05407 |
| Smoker (<20 packs of cigarettes/year />=20 packs of cigarettes/year) | 1.02 | 0.74-1.4 | 0.908 | | 1.4 | 0.88-2.22 | 0.15948 |
| ECOG (0/1/2) | 1.25 | 0.82-1.91 | 0.3044 | | 1.03 | 0.65-1.64 | 0.90037 |
| Pathology (squamous cell carcinoma/adenocarcinoma) | 0.85 | 0.63-1.14 | 0.2734 | | 0.72 | 0.5-1.03 | 0.07126 |
| Brain metastases (Yes/No) | 1.18 | 0.79-1.77 | 0.4229 | | 1.34 | 0.74-2.42 | 0.33302 |
| Liver metastases (Yes/No) | 1.08 | 0.72-1.62 | 0.7078 | | 1.23 | 0.8-1.89 | 0.3467 |
| Thoracic Radiotherapy (Yes/No) | 1.31 | 0.96-1.78 | 0.0874 | | 1.23 | 0.86-1.76 | 0.25556 |
| Brain Radiotherapy (Yes/No) | 0.99 | 0.57-1.71 | 0.9611 | | 0.61 | 0.28-1.32 | 0.20971 |
| Lines of ICI therapy (1/2/3) | 1.04 | 0.74-1.44 | 0.8297 | | 0.82 | 0.56-1.21 | 0.31487 |
| Evaluation of efficacy (PR/SD) | 1.48 | 1.08-2.02 | 0.0155 | | 1.68 | 1.16-2.43 | 0.00564 |
| Immunotherapy (monotherapy/combination) | 0.77 | 0.56-1.06 | 0.1063 | | 0.73 | 0.51-1.05 | 0.08839 |
| Overall survival |  |  |  | |  |  |  |
| Age (≥65/<65) | 1.24 | 0.82-1.87 | 0.3141 | | 1.25 | 0.78-1.99 | 0.3505 |
| Sex (Male/Female) | 1.51 | 0.86-2.63 | 0.1512 | | 2.06 | 0.99-4.26 | 0.0518 |
| Smoker (<20 packs of cigarettes/year />=20 packs of cigarettes/year) | 1.03 | 0.68-1.58 | 0.8792 | | 1.08 | 0.59-1.95 | 0.8073 |
| ECOG (0/1/2) | 1.27 | 0.71-2.28 | 0.422 | | 1.24 | 0.66-2.33 | 0.5078 |
| Pathology (squamous cell carcinoma/adenocarcinoma) | 0.77 | 0.52-1.14 | 0.1919 | | 0.51 | 0.32-0.82 | 0.0049 |
| Brain metastases (Yes/No) | 0.63 | 0.33-1.22 | 0.1732 | | 0.74 | 0.33-1.66 | 0.4599 |
| Liver metastases (Yes/No) | 1.08 | 0.62-1.87 | 0.7881 | | 1.09 | 0.61-1.95 | 0.7829 |
| Thoracic Radiotherapy (Yes/No) | 1.26 | 0.83-1.9 | 0.2814 | | 1.02 | 0.64-1.64 | 0.9274 |
| Brain Radiotherapy (Yes/No) | 0.34 | 0.11-1.07 | 0.0647 | | 0.27 | 0.07-1.06 | 0.0597 |
| Lines of ICI therapy (1/2/3) | 0.86 | 0.55-1.33 | 0.495 | | 0.79 | 0.48-1.31 | 0.3652 |
| Evaluation of efficacy (PR/SD) | 1.42 | 0.92-2.17 | 0.1101 | | 1.9 | 1.15-3.12 | 0.0118 |
| Immunotherapy (monotherapy/combination) | 0.67 | 0.44-1.01 | 0.0569 | | 0.55 | 0.34-0.88 | 0.0139 |

**Table s2** Univariate and multivariate analyses of disease progression model on Progression-Free Survival-2 and Overall Survival (Cox regression) (N=208)

| Factor | Univariate analysis | | | Multivariate analysis | | |
| --- | --- | --- | --- | --- | --- | --- |
|  | **HR (log rank)** | **95% CI** | **p value** | **HR (log rank)** | **95% CI** | **p value** |
| Progression-Free Survival-2 |  |  |  |  |  |  |
| Oligo-progression/Systemic progression | 1.72 | 1.28-2.32 | <0.001 | 1.72 | 1.28-2.32 | <0.001 |
| Overall survival |  |  |  |  |  |  |
| Oligo-progression/Systemic progression | 1.87 | 1.26-2.77 | 0.0018 | 1.87 | 1.26-2.77 | 0.0018 |

**Table s3** Univariate and multivariate analyses of treatment strategy after disease progression from ICI on Progression-Free Survival-2 and Overall Survival (Cox regression) (N=208)

| Factor | Univariate analysis | | | Multivariate analysis | | |
| --- | --- | --- | --- | --- | --- | --- |
|  | **HR (log rank)** | **95% CI** | **p value** | **HR (log rank)** | **95% CI** | **p value** |
| Progression-Free Survival-2 |  |  |  |  |  |  |
| 0 |  |  |  |  |  |  |
| 1 | 0.6 | 0.3-1.18 | 0.1357 | 5.91 | 1.47-23.72 | 0.0122 |
| 2 | 1.03 | 0.57-1.87 | 0.9128 | 1.18 | 0.53-2.66 | 0.6853 |
| 3 | 0.48 | 0.29-0.78 | 0.0032 | 0.22 | 0.06-0.89 | 0.0336 |
| 1+2 | 0.34 | 0.1-1.13 | 0.0785 | 4.18 | 0.71-24.53 | 0.1135 |
| 1+3 | 0.21 | 0.09-0.49 | <0.001 | 0.96 | 0.13-6.81 | 0.9643 |
| 1+4 | 0.38 | 0.22-0.65 | <0.001 | 3.25 | 0.99-10.64 | 0.0510 |
| 2+4 | 0.32 | 0.19-0.53 | <0.001 | 0.35 | 0.19-0.63 | 0.0005 |
| 3+4 | 0.63 | 0.34-1.2 | 0.1588 | 0.28 | 0.07-1.22 | 0.0903 |
| 1+2+4 | 0.18 | 0.09-0.4 | <0.001 | 2.23 | 0.49-10.12 | 0.3000 |
| ICI halt | 0.6 | 0.45-0.81 | <0.001 | NA | NA | NA |
| ICI maintain |  |  |  |  |  |  |
| Anti-angiogenic (yes) | 0.57 | 0.41-0.78 | <0.001 | 0.1 | 0.03-0.33 | 0.0002 |
| Anti-angiogenic (no) |  |  |  |  |  |  |
| radioation therapy (yes) | 0.75 | 0.55-1.04 | 0.0812 | 0.81 | 0.45-1.45 | 0.4773 |
| radioation therapy (no) |  |  |  |  |  |  |
| Chemotherapy (yes) | 1.02 | 0.74-1.4 | 0.8963 | 2.17 | 0.58-8.17 | 0.2531 |
| Chemotherapy (no) |  |  |  |  |  |  |
| Overall survival |  |  |  |  |  |  |
| 0 |  |  |  |  |  |  |
| 1 | 0.52 | 0.23-1.18 | 0.1195 | 4.23 | 0.93-19.32 | 0.0626 |
| 2 | 1.17 | 0.61-2.23 | 0.6434 | 1.65 | 0.65-4.21 | 0.2974 |
| 3 | 0.25 | 0.13-0.47 | <0.001 | 0.21 | 0.04-1.22 | 0.0824 |
| 1+2 | 0 | 0-Inf | 0.9951 | 0.00 | 0-Inf | 0.9958 |
| 1+3 | 0.08 | 0.02-0.34 | <0.001 | 0.56 | 0.05-7.06 | 0.6572 |
| 1+4 | 0.27 | 0.14-0.53 | <0.001 | 1.73 | 0.52-5.74 | 0.3706 |
| 2+4 | 0.16 | 0.08-0.31 | <0.001 | 0.19 | 0.09-0.4 | 0.0000 |
| 3+4 | 0.26 | 0.1-0.68 | 0.0063 | 0.22 | 0.03-1.48 | 0.1189 |
| 1+2+4 | 0.07 | 0.02-0.24 | <0.001 | 0.78 | 0.12-5.14 | 0.8004 |
| ICI halt | 0.44 | 0.29-0.66 | <0.001 | NA | NA | NA |
| ICI maintain |  |  |  |  |  |  |
| Anti-angiogenic (yes) | 0.5 | 0.31-0.79 | 0.0028 | 0.11 | 0.03-0.41 | 0.0009 |
| Anti-angiogenic (no) |  |  |  |  |  |  |
| Radioation therapy (yes) | 0.78 | 0.51-1.2 | 0.2567 | 0.66 | 0.31-1.42 | 0.2884 |
| Radioation therapy (no) |  |  |  |  |  |  |
| Chemotherapy (yes) | 0.67 | 0.42-1.06 | 0.0904 | 1.08 | 0.21-5.62 | 0.9246 |
| Chemotherapy (no) |  |  |  |  |  |  |

0, best supportive care; 1, anti-angiogenesis; 2, local radiotherapy; 3, chemotherapy; 4, ICI maintain
